# Unveiling the neglected role of the intensity of acute stress disorder in the prediction of full- and sub-threshold posttraumatic stress disorder: looking beyond the diagnosis

**DOI:** 10.1007/s00127-024-02805-z

**Published:** 2024-12-31

**Authors:** Elie G. Karam, Josleen Al Barathie, Hani Dimassi, Franco Mascayano, Andre Slim, Aimee Karam, George Karam, Katherine M. Keyes, Ezra Susser, Richard Bryant

**Affiliations:** 1 Institute for Development, Research, Advocacy and Applied Care (IDRAAC), Beirut, Lebanon; 2 Department of Psychiatry and Clinical Psychology, University of Balamand Faculty of Medicine, Beirut, Lebanon; 3 Department of Psychiatry and Clinical Psychology, St George Hospital University Medical Center, Beirut, Lebanon; 4 School of Pharmacy, Lebanese American University, Beirut, Lebanon; 5 Department of Epidemiology, Columbia University Mailman School of Public Health, New York, NY USA; 6 New York State Psychiatric Institute, New York, NY USA; 7 School of Psychology, University of New South Wales, Sydney, NSW 2052 Australia; 8 P.O. Box: 166227, Ashrafieh, Beirut, 1100 2110 Lebanon

**Keywords:** Trauma, Blast exposure, Prospective studies, Disasters, Mental health, Medical personnel

## Abstract

**Purpose:**

Exposure to traumatic events may lead to the development of Acute Stress Disorder (ASD) within the first month post-trauma in some individuals, while others may not exhibit ASD symptoms. ASD was introduced as a potential early indicator to identify those at higher risk of developing Posttraumatic Stress Disorder (PTSD), however, PTSD can occur in some individuals even without prior ASD. Assessing ASD post-trauma can assist in identifying those who would most benefit from intervention to prevent later PTSD, yet the predictive power of ASD varies across studies, with intensity of ASD symptoms and subthreshold PTSD often less considered.

**Methods:**

A prospective cohort study on 426 health workers exposed to the Beirut Port Blast assessed DSM-5 ASD and symptom intensity using self-report questionnaire at two distinct time points: 9–15 and 21–27 days post blast. DSM-5 PTSD was assessed afterwards at 6–7 months via self-report questionnaire post-exposure. Probit models predicted full and subthreshold PTSD.

**Results:**

Using ASD diagnosis alone, the sensitivity 9–15 days after trauma was better than 21–27 days after trauma (75.68% vs. 58.06%); when stratified by intensity, however, sensitivity increased from 41.66% among those with low intensity to 92% among those with high intensity. Specificity, however, was better 21–27 days after trauma (77.82%) compared with 9–15 days (60.98%). Positive Predictive Value of ASD increased, and Negative Predictive Value decreased, with time since exposure and when adding intensity with diagnosis. ASD diagnosis plus intensity achieved better prediction of PTSD and subthreshold PTSD.

**Conclusion:**

Screening for PTSD should include ASD and its intensity, improving predictive ability for later PTSD, incorporatingfull threshold and subthreshold PTSD. Specificity increases with time since exposure, suggesting a high rate of false positives when assessing ASD soon after trauma. This highlights the need to prioritize individuals for early preventive measures after trauma.

**Supplementary Information:**

The online version contains supplementary material available at 10.1007/s00127-024-02805-z.

## Introduction

The mental health consequences of trauma are known to be frequent and varied, the most immediate being Acute Stress Disorder (ASD) and one or more months later posttraumatic stress disorder (PTSD) [1]. Traumatic events also increase the risk of depression, anxiety disorders, substance use, and suicide [2].

ASD was initially introduced in the Diagnostic and Statistical Manual of Mental Disorders the fourth edition (DSM-IV) [3] to describe severe posttraumatic stress reactions in the initial month after trauma exposure. ASD was also proposed to identify individuals within the first month post-trauma who are at risk of developing subsequent PTSD [4]. DSM-5 introduced several modifications to the criteria defining ASD [5]. DSM-5 required at least 9 out of 14 symptoms to reach a diagnosis, all of equal importance, and the revised diagnosis was reported to have better prediction of subsequent PTSD and other psychiatric disorders than DSM-IV [6]. The predictive power of PTSD by ASD has been evaluated systematically in a review of 22 studies which found that DSM-IV ASD has a range of positive predictive values (PPVs) from 14 to 82% and negative predictive values (NPVs) from 65 to 96%, with sensitivities from 20 to 72% and specificities from 56 to 97% [7]. PPV indicates the proportion of individuals diagnosed with ASD who subsequently develop PTSD, highlighting that a high PPV suggests a strong likelihood of PTSD in those diagnosed with ASD, while a low PPV indicates that many diagnosed with ASD may not develop PTSD. Conversely, NPV measures the proportion of individuals not diagnosed with ASD who do not go on to develop PTSD; a high NPV indicates that most individuals without ASD will not develop PTSD, but a low NPV suggests that PTSD can still occur in individuals without prior ASD diagnosis. These interpretations emphasize the complexity of the relationship between ASD and PTSD, reminding us that not everyone with ASD will develop PTSD and not all those without ASD will be free from risk. A more recent review found similar results with a wide range of PPVs and NPVs, sensitivities and specificities with a tendency of those using DSM-5 to yield somewhat better results [8]. One study that focused on timing of assessment of ASD, a rarely researched issue, found that ASD prediction of PTSD was improved when ASD was assessed 4 weeks after the trauma rather than immediately after the trauma [9], yet predictive values were not reported. Finally, one study reported that combining ASD diagnosis and its intensity could better predict later PTSD following bank robbery [10].

In addition, an important line of research has investigated subthreshold PTSD, defined as meeting most but not all required symptom criteria to meet PTSD diagnosis. One multinational study comprising 13 countries and involving 23,936 respondents demonstrated that many trauma survivors report subthreshold levels of PTSD but nonetheless experience marked distress or impairment [11]. This finding accords with other studies attesting to the impairment association with subthreshold PTSD [12, 13]. Recognizing these symptoms allows healthcare providers to address the needs of individuals who may otherwise go unnoticed or untreated. Addressing subthreshold symptoms early can also improve long-term mental health outcomes and quality of life, as individuals with subthreshold PTSD may experience chronic symptoms leading to functional impairment and increased risk for comorbid conditions such as depression and anxiety. These findings indicate that the potential capacity of ASD to predict subthreshold PTSD is as important as predicting full threshold PTSD diagnosis.

A meta-analysis of secondary prevention of PTSD suggested that treating ASD before the emergence of PTSD offers true possibilities of prevention of post-trauma symptoms [14]. To further evaluate this possibility, it is essential that additional research address the predictive value of timing of ASD, combination of ASD and intensity, and sub-threshold PTSD in order to identify those at highest risk for PTSD after trauma. Expanding resources for early intervention (time, human effort, and money) needs to focus on trauma victims most likely to later suffer from PTSD or clinically significant subthreshold PTSD.

In Beirut, Lebanon, a significant recent population-wide trauma was an explosion in a major city port in 2020 (“Beirut Post Blast”). This event led to deaths, thousands of injuries, and displacement in the Beirut population; the consequences of exposure on population-wide mental health is thus of critical public health significance and it offers an opportunity to study the mental health consequences of trauma on a large scale. The present study following the Blast of Beirut Port in 2020 aims to evaluate the capacity of ASD, assessed less than one month after the blast, to predict PTSD 6–7 months post blast. Importantly, we hypothesize that (1) the assessment of not only categorical ASD diagnosis is important, but also the intensity of ASD increases PTSD predictiveness (2) the interval post-trauma at which ASD was measured will affect the predictive power (3) ASD symptoms will serve as a strong predictor of both full and subthreshold DSM-5 PTSD.

## Methods

### Study design, setting, and participants

On the 4th of August 2020, approximately 2750 metric tons of ammonium nitrate stored in the Port of Beirut (Lebanon) exploded, producing one of the largest non-nuclear explosions in modern history [15, 16]. The explosion left 217 fatalities, 6,000 injured, and approximately 300,000 displaced families [17]. One of the most severely affected edifices by the Beirut Port Blast was the Saint George Hospital University Medical Center (SGHUMC), due to its proximity to the Port [18]. The staff at SGHUMC, at the forefront, witnessed deaths, mutilation, destruction, and chaos, rendering them front line victims important to follow prospectively to monitor the effects of the blast. Three waves of data collection have been conducted so far at 9–15 days, 21–27 days and 6–7 months post Beirut Port Blast. The first two waves were a national initiative following the Beirut Port Blast while the third wave was also part of an ongoing longitudinal multi-national study focused on health care workers and COVID-19 [19]. In wave 1, data was collected face-to-face 9–15 days post blast (further details about the data collection process and the response rate are present elsewhere [18] and participants provided oral informed consent to participate in the research. Waves 2 and 3 were collected through an online survey and were performed 21–27 days and 6–7 months post blast, respectively. This data was collected online by disseminating survey links through mass emails to the hospital’s employees (further details in Online Resource 1). In those waves, informed written consent was taken from participants before they started filling the online surveys. These consents were later emailed to the respective participants for full transparency.

The study participants were health workers at SGHUMC aged 18 years and older employed in clinical and administrative departments, as well as support staff (e.g., in food and environmental services). Clinical professionals include nurses, doctors, residents, laboratory technicians, radiologists, dietitians and other health-care workers, whereas non-clinical professionals include admission and patient’s account, budget department, environmental services, finances, information desk, general services, human resources, medical engineering, material management, laundry and other administrative and services staff. The total number of workers at SGHUMC was 1,927 in waves 2 and 3. The total number of respondents was 733 and 808 which correspond to a response rate of 38.0% and 41.8% respectively. In wave 3, out of the total number of 808 respondents, we have information of 426 participants (50 exclusively followed up from wave 1; 209 exclusively followed up from wave 2; and 167 from waves 1 and 2). Online Resource 1 provides a more detailed assessment of participants by wave. We focus the present study on the 426 individuals who responded on the Beirut Port Blast Exposure Inventory and acute trauma questionnaire in Waves 1 and/or 2 and completed follow-up in Wave 3 where we assessed PTSD secondary to the blast as well as other prior and present mental health disorders, in addition to previous trauma and other psychosocial variables. Since this study focuses on those who are followed up, waves 1 and 2 featured short, 10-minute survey. Afterwards, we followed those people who filled the survey up and asked them to participate in wave 3. Although wave 3 was longer, yet key measures such as the PCL-5 were placed immediately after the sociodemographic questions, ensuring full completion by the participants and minimizing the likelihood of item-level missing responses.

This study was approved by the Institutional Review Board (IRB) committee of the SGHUMC Faculty of Medicine, University of Balamand, Lebanon, which is registered with the U.S Office of Human Research Protections (OHRP) in the Department of Health and Human Services.

### Measures

The Acute Stress Disorder Scale (ASDS) [20] was used in waves 1 and 2 and the PTSD Checklist for DSM-5 (PCL-5) was used in Wave 3 [21].

#### Acute stress disorder scale

We measured the ASD in waves 1 and 2 using the ASDS. A previous study that compared the ASDS with the gold standard Acute Stress Disorder Interview (ASDI) for DSM-IV reported a sensitivity of 0.95, specificity of 0.83, PPV of 0.80, and NPV of 0.96 [20]. The ASDS adapted for DSM-5 consists of 20 items, 19 of which ask about symptoms of acute stress following trauma. Each of these 19 items is coded on a 5-point scale (1 = not at all, 5 = very much) and the summation of scores provides a total intensity score [22]. Participants were also asked if they experienced fear during the blast (item 20) [20]. The original English ASDS scale was translated to Arabic and showed an excellent internal consistency of 0.94. Probable DSM-5 ASD was defined as endorsing at least 9 of the 14 DSM-5-relevant symptoms.

#### Posttraumatic stress disorder scale

We assessed PTSD using the PCL-5, which is a well-established 20-item self-report measure of the 20 DSM-5 symptoms of PTSD [21]. Each symptom is rated from 0 to 4 scale (0 = n*ot at all*, 4 = e*xtremely*). The DSM-5 diagnostic rule requires that a person meets at least 1 B criterion item (intrusion), 1 C criterion item (persistent avoidance), 2 D criteria items (negative alterations in cognitions and mood), and 2 E criteria items (arousal and reactivity). For the subthreshold DSM-5 definition of PTSD, two definitions were used in which (a) at least six symptoms of PTSD were present (‘Six Plus’), and (b) full threshold for three of the four B-E criteria (‘Majority’) [11]. The original English PCL-5 scale was translated to Arabic by members of our team and back-translated to English by a professional translator.

In addition to the primary variables of interest, participant characteristics such as age, gender, and profession were also collected.

### Statistical analysis

Given the nature of the online survey, where participants’ responses were directly recorded with restriction on continuous variables such as age, there was minimal risk of data entry misclassification errors. Nevertheless, during data management, we carefully examined all variables to ensure accuracy. Quality control checks were conducted after each computation, verifying the correctness of summations and diagnostic categorizations. These steps ensured the dataset was clean and ready for further analysis.

Descriptive statistics were used to summarize sample characteristics. For continuous variables, means and standard deviations (SD) were reported, while categorical variables were presented as frequencies and percentages (N, %). The analyses included the 426 participants who were followed up from Waves 1 and/or 2 and Wave 3.

The prevalence of ASD in Waves 1 and 2, and the prevalence of both fullthreshold and subthreshold PTSD in Wave 3 were reported. ASD symptom severity is presented in Tables 1 and 2 and the supplementary tables corresponding to the different survey waves in this manuscript.

Analyses of the current study were performed using Stata/MP 13. The prediction of full threshold and subthreshold PTSD was performed using Probit models in two ways: (1) using ASD diagnosis only and (2) using the ASDS total score (to account for intensity) over and above the diagnosis of ASD.

The predictive abilities were undertaken using NPV (NPV: the probability that following a negative ASD diagnosis, that individual will not develop PTSD), PPV (PPV: the probability that following a positive ASD diagnosis, that individual will develop PTSD), sensitivity (the proportion of people who test positive for ASD among all those who develop PTSD) and specificity (the proportion of people who test negative for ASD among all those who develop PTSD).

A Receiver Operating Characteristic curve (ROC) with an Area Under the Curve (AUC) and sensitivity analyses were conducted to check for the optimal cut-off score of the ASDS prediction of PTSD. One earlier study of the ASDS found that a score of 56 optimally predicted later PTSD [20]. We found a score of 58 generated a slightly better results than 56 in our population (Youden’s index: 0.361 vs. 0.357). The cut-off of 58 produced an AUC of 0.705 (*p* < 0.001), demonstrating acceptable discriminatory power and was used in our analyses.

## Results

### Sample characteristics

Of the 426 participants followed up, the mean age was 36.31 years (SD = 12.03), with a range of 20 to 73 years. The majority were female (*N* = 324, 76.06%). In terms of profession, most of the participants were clinical professionals (*N* = 318, 74.65%).

### ASD prevalence (9–15 and 21–27 days after trauma)

In the analytic sample, the prevalence of ASD 9–15 days after trauma was 45.8% (*N* = 92 out of 201) and 21–27 days after trauma was 31.3% (*N* = 115 out of 368).

### PTSD prevalence (6–7 months after trauma)

At 6–7 months post-trauma, fullthreshold PTSD prevalence was 29.6% (*N* = 221). In terms of the “Majority” subthreshold definition of PTSD, the prevalence of subthreshold combined with full threshold DSM-5 PTSD was 48.3% (*N* = 362); using the “Six Plus” definition, the prevalence of full threshold combined with subthreshold PTSD was 51.9% (*N* = 389). Combining the three definitions, the prevalence of full/ subthreshold PTSD was 53.9%.

### Prediction of PTSD

#### Positive predictive value

PPV was assessed for both 9–15 days after trauma and 21–27 days after trauma. Both ASDS diagnosis and its intensity (below vs. above the cut-off of 58 and a continuous score) were used to predict PTSD 6–7 months later. When DSM-5 ASD diagnosis is used alone in predicting DSM-5 PTSD diagnosis 6–7 months after trauma, the PPV was 30.4% in 9–15 days after trauma and 47.0% 21–27 days after trauma (Table 1).

We also stratified the sample into those who have both DSM-5 ASD and high versus low intensity, based on a cut-off of 58. Among those with low intensity ASD, PPV 9–15 days after trauma was 22.7% and 32.3% 21–27 days after trauma. Among those with high intensity, PPV 9–15 days after trauma was 32.9% while 21–27 days after trauma was 53.1% (Table 1).

**Fig. 1:**
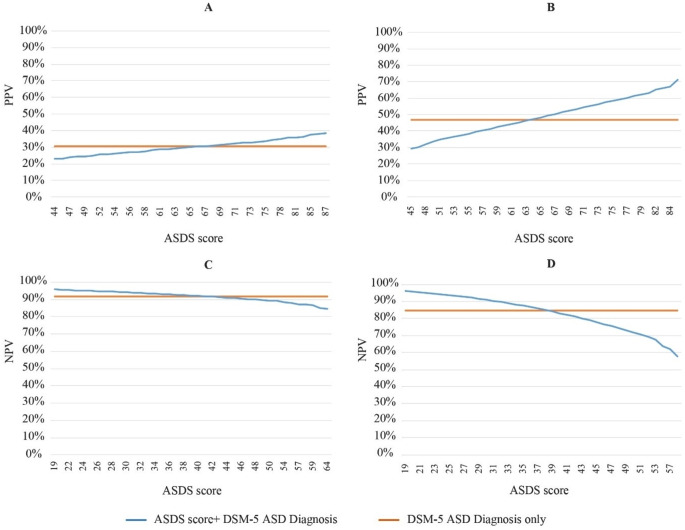
Variation of Positive Predictive Value (PPV) of ASD 9–15 days after trauma (**A**) and 21–27 days after trauma (**B**), and Variation of Negative Predictive Value (NPV) of ASD at 9–15 days after trauma (**C**) and 21–27 days after trauma (**D**) predicting the outcome of full threshold DSM-5 PTSD diagnosis 6–7 months after trauma. PPV: Positive Predictive Value; NPV: Negative Predictive Value; ASDS: Acute Stress Disorder Scale. This figure is a 2-column fitting image /colored

When ASDS score was added as a screener over and above DSM-5 ASD diagnosis, as the ASDS score increases, the PPV ranged then from 23.0 to 38.3% 9–15 days after trauma (Fig. 1-A) and from 29.2 to 71.3% 21–27 days after trauma (Fig. 1-B).

#### Negative predictive value

Using ASD diagnosis as a predictor of PTSD, NPV was 91.7% 9–15 days after trauma and 84.6% 21–27 days after trauma (Table 1). When stratified by intensity, NPV 9–15 days after trauma dropped from 93.1% when ASDS was below the cut-off to 71.4% when ASDS was above the cut-off, while 21–27 days after trauma, it dropped from 85.1 to 60.0% (Table 1).

**Table 1:** Negative predictive value (NPV) and positive predictive value (PPV) of ASD 9–15 days, and 21–27 days, after trauma predicting PTSD 6–7 months after trauma: variations in total sample, full threshold DSM-5 PTSD diagnosis

	*N*	Any DSM-5 ASD Diagnosis	DSM-5 ASD Diagnosis *and* ASDS Score	
			Low intensity	High intensity
NPV 9–15 days after trauma	109	91.74	93.14	71.43
PPV 9–15 days after trauma	92	30.43	22.73	32.86
NPV 21–27 days after trauma	253	84.58	85.08	60
PPV 21–27 days after trauma	115	46.96	32.35	53.09

^* High/low intensity defined as cut−off above or below 58^

Adding ASDS intensity over and above ASD diagnosis, NPV ranged from 84.8% (maximum ASDS score) to 95.9% (minimum ASDS score). The NPVs across the full range of scores 9–15 days after trauma can be seen in Fig. 1-C. For 21–27 days after trauma, the NPV ranged from 57.6% (maximum ASD score) to 96.1% (minimum ASD score). The NPVs across the full range of scores 21–27 days after trauma are presented in Fig. 1-D.

#### Sensitivity

Using ASD diagnosis as the sole predictor, the sensitivity 9–15 days after trauma was better than 21–27 days after trauma (75.68% vs. 58.06%).

When stratified by intensity, sensitivity increased from 41.7% among those with low intensity to 92.0% among those with high intensity (Table 2). At 21–27 days after trauma, the sensitivity increased from 26% among those with low intensity to 95.3% among those with high intensity.

**Table 2:** Sensitivity and specificity of ASD 9–15 days, and 21–27 days, after trauma and PTSD 6–7 months later: variations in total sample, full threshold DSM-5 PTSD diagnosis

	*N*	Any DSM-5 ASD Diagnosis	DSM-5 ASD Diagnosis *and* ASDS Score	
			Low intensity	High intensity
Sensitivity: 9–15 days after trauma	37	75.68	41.66	92
Specificity: 9–15 days after trauma	164	60.98	84.82	9.62
Sensitivity 21–27 days after trauma	93	58.06	26	95.35
Specificity: 21–27 days after trauma	275	77.82	89.79	7.5

^* High/low intensity defined as cut−off above or below 58^

#### Specificity

Unlike sensitivity, specificity was better 21–27 days after trauma than 9–15 days after trauma (77.8% vs. 61.0%) (Table 2). When considering the intensity of ASD over and above ASD diagnosis, specificity was very low among those with high intensity (9.6% 9–15 days after trauma and 7.5% 21–27 days after trauma) compared to those with low intensity (84.8% 9–15 days after trauma and 89.8% 21–27 days after trauma) (Table 2).

### Prediction of full and subthreshold PTSD

Similar trends to the pattern in predicting PTSD, the addition of the ASDS score over and above ASD diagnosis improved the prediction of full/subthreshold PTSD in which PPV increased to 79.5% 21–27 days after trauma using the ‘Majority’ and ‘82.2%’ for ‘Six Plus’ definitions (see Online Resource 2). Specificity improved up to 93.6% 21–27 days after trauma for the ‘Majority’ and 93.5% for the ‘Six Plus’ (see Online Resource 3).

No clear improvement in NPV and sensitivity was noticed when looking at full/subthreshold PTSD prediction using ASD compared to full PTSD. This was evident because the NPV had reached a maximum of 79.6% for ‘Majority’ and 80.6% for ‘Six Plus’ in 9–15 days after trauma compared to 95.9% with full threshold alone (See Online Resource 2). Sensitivity has reached 96.6% 21–27 days after trauma for ‘Majority’ and 96.7% 21–27 days after trauma for ‘Six Plus’ which is approximately equal to 96.1% for full threshold alone (see Online Resource 3). The variations of NPV and PPV 9–15 and 21–27 days after trauma across different ASDS scores can be found in Online Resource Figs. 1 and 2. It is worth noting that the trends of subthreshold and full threshold look similar to the full threshold alone; as the ASDS increases, NPV decreases and PPV increases.

## Discussion

Identifying recently trauma-exposed people who are at highest risk of subsequent PTSD is a key clinical and public health priority. Early interventions to prevent PTSD have been validated [23, 24] and are recommended in most treatment guidelines [25, 26]. The current study advances knowledge about early detection of people who will develop PTSD by considering the different metrics of ASD diagnosis and intensity in predicting full and subthreshold PTSD.

Our data show that intensity scores for acute disorder should be considered over and above diagnosis of ASD. Adding intensity to the ASD diagnosis, as measured by the ASDS, improved the predictive power of ASD to a large degree. For example, 21–27 days after trauma, PPV increased from 29.2 to 71.3% and sensitivity increased from 26.0 to 95.3%. For public health purposes, while the ASD diagnosis can be helpful, it may not have the same predictive power without including the intensity of the acute stress response. We thus recommend that health professionals first assess the presence of the ASD diagnosis and then evaluate its intensity. Those with both ASD and with high intensity ASD are at high risk of subsequent PTSD.

Our findings demonstrate that the intensity of acute stress reactions plays a crucial role, even among individuals who do not meet the criteria for ASD but later develop PTSD. For example, while 76% of individuals without ASD diagnosis did not develop PTSD (as indicated by the NPV in the majority subthreshold group), when intensity of the acute stress response is considered, the predictive value changes significantly. Among those without ASD but with highintensity acute stress, the NPV drops to 28.57%, indicating that 71.43% of these individuals went on to develop PTSD even with no ASD diagnosis. This underscores the importance of not relying solely on the presence of an ASD diagnosis, as doing so may overlook a substantial number of people at risk. Incorporating both the presence and intensity of acute stress responses allows for a more accurate prediction of PTSD and provides an opportunity for timely intervention.

On the basis that significant functional impairment, poor quality of life and increased risk of comorbid conditions are associated with subthreshold PTSD [11], it is noteworthy that we were able to reasonably predict the presence of at least subthreshold PTSD (as defined by both the ‘Six Plus’ and the ‘Majority’ methods). Restricting prediction to full threshold PTSD may neglect a significant group of people who will experience significant distress and impairment associated with subthreshold levels [13], and so the observation that ASD combined with acute stress intensity can predict people with at least subthreshold PTSD has the potential to facilitate identification of people who can benefit from early intervention, recognize their distress early on, and foster a comprehensive approach to mental health care following trauma exposure. This capacity is particularly relevant in the context of evidence that in the years after trauma exposure many people oscillate between full and subthreshold levels of PTSD [27].

In terms of the timing of ASD assessment, NPV and sensitivity were better 9–15 days after trauma, whereas PPV and specificity were better 21–27 days after trauma. This is an interesting finding because it suggests that if one wishes to predict the greatest proportion of people who will develop full/ subthreshold PTSD, it may be better to assess people sooner after trauma exposure. In contrast, if one is wanting to achieve greater accuracy (and fewer false positive identifications), then the assessment may function better when it is delayed. This pattern of findings can be considered in light of the different predictive roles of certain ASD symptoms according to when they are assessed. For example, studies have found that persistent dissociation in the month after trauma exposure are more predictive of later PTSD than when peritraumatic dissociation occurring during the trauma itself [9, 28]. Finally, it is possible that in future definitions of ASD, the dimensional aspect should be taken into consideration, mirroring the scores of ASDS [12], and enhancing the prediction of full-blown PTSD [29].

## Limitations

First, the findings of our study are based on a sample of healthcare workers from one hospital; hence, they may not generalize to non-health care workers. Second, it is not known whether these results could be generalized to all types of traumas. Hence, replication in another setting with other types of traumas is an important future direction. Third, a well-known drawback of cohort studies is the loss to follow-up; in spite of all our efforts, we were not able to follow all subjects, and this might have affected our results. Fourth, of course there are other factors that affect the emergence of PTSD and other subthreshold post-traumatic syndromes, including previous traumas, other mental health disorders, and other stressors (past and present). Along the same lines, an important factor that affects PTSD endorsement, which we did not inquire about, is whether participants had received treatment at the 6–7 month assessment. This should be considered when interpreting our findings and represents a limitation of our study where future research would benefit from including questions about treatment history to enhance the understanding of its potential impact on PTSD outcomes. Gains have been made in recent years in integrating various acute variables into predictive models, which can potentially improve early identification of people who will develop PTSD [30, 31]. Fifth, we did not use a clinical face-to-face diagnosis for ASD and PTSD. Instead, we have used the self-reported ASDS and PCL-5 to assess ASD and PTSD respectively. However, the sensitivity and specificity of ASDS, is proven to be quite effective in identifying ASD. In addition, PCL-5 has been found to be a valid measure in assessing PTSD. Furthermore, in many global settings where significant trauma has occurred, self-report scales are the optimal method of assessing population mental health given the low density of clinicians in many emergency situations. Finally, in addition to ASD symptoms, several other factors could be instrumental in predicting worsening symptoms after trauma exposure, which our study did not consider. For instance, Galatzer-Levy et al. [32] emphasize that individuals show diverse responses to significant life stressors and potential trauma, with resilience being the most prevalent outcome that influences trauma reactions. Additionally, the accumulation of trauma experiences and continuous victimization has been shown to significantly contribute to the progressive worsening of PTSD symptoms [33]. Moreover, comorbid conditions like depression are critical in shaping the trajectory of PTSD symptoms, as evidenced by longitudinal studies involving U.S. service members and veterans, where the co-occurrence of PTSD and depression intensified symptom severity and hindered recovery [34]. Other factors, such as a lack of social support and pre-existing mental health issues, also play a significant role. Recognizing and evaluating these additional elements can enhance predictive models and lead to more effective targeted interventions for individuals at risk.

## Conclusion

ASD, and crucially its intensity, has reasonable predictive values of later PTSD, including not only full threshold PTSD but also its subthreshold. This is of central importance to help predicting which section of the population would need preventive measures early on after trauma. Finally, to limit overestimation of future PTSD, it is preferable to delay assessment till several weeks after the trauma.

## Electronic supplementary material

Below is the link to the electronic supplementary material.

Supplementary Material 1

Supplementary Material 2

Supplementary Material 3

Supplementary Material 4

## Data Availability

The dataset generated for this research is available in the Figshare repository, https://figshare.com/ on this URL: 10.6084/m9.figshare.28015220.v1.
