# Supplementary material for: Unveiling the neglected role of the intensity of acute stress disorder in the prediction of full- and sub-threshold posttraumatic stress disorder: looking beyond the diagnosis
Source: Soc Psychiatry Psychiatr Epidemiol. 2024 Dec 31;60(5):1125–33. doi: 10.1007/s00127-024-02805-z (PMC12119768; doi:10.1007/s00127-024-02805-z)
Supplement: Supplementary file 1 — Supplementary Material 1 [file 127_2024_2805_MOESM1_ESM.docx]

**Title:** Unveiling the Neglected Role of the Intensity of Acute Stress Disorder in the Prediction of full- and sub-threshold posttraumatic stress disorder: Looking Beyond the Diagnosis.

**Journal of Social Psychiatry and Psychiatric Epidemiology**

**Authors names and affiliations:** Elie G. Karam^a,b,c^. Josleen Al Barathie^a^, Hani Dimassi^d^, Franco Mascayano^e,f^, Andre Slim^a^, Aimee Karam^a,b,c^, George Karam^a,b,c^, Katherine M. Keyes^e^, Ezra Susser^e,f^, Richard Bryant^h^.

a Institute for Development, Research, Advocacy and Applied Care (IDRAAC), Beirut, Lebanon

b Department of Psychiatry and Clinical Psychology, University of Balamand Faculty of Medicine, Beirut, Lebanon

c Department of Psychiatry and Clinical Psychology, St George Hospital University Medical Center, Beirut, Lebanon

d School of Pharmacy, Lebanese American University, Beirut, Lebanon

e Department of Epidemiology, Columbia University Mailman School of Public Health, New York, NY, United States

f New York State Psychiatric Institute, New York, NY, United States

h School of Psychology, University of New South Wales, NSW 2052, Sydney, Australia

**Corresponding Author:**

Email: [egkaram@idraac.org](mailto:egkaram@idraac.org)

Supplementary Table 1: Distribution of participants across different waves

|  | Wave 1 | Wave 2 | Wave 3 |
| --- | --- | --- | --- |
| Time of assessment (post blast) | 9-15 days | 21-27 days | 6-7 months |
| Mode of assessment | Face to face: self-filled questionnaire in offices next to PCR screening area | Mass emails sent via MailChimp + SMS and WhatsApp reminders: self-filled questionnaire online | Mass emails sent via MailChimp + SMS and WhatsApp reminders + Personalized letters with QR codes: self-filled questionnaire using Chilean Platform |
| Total Number of participants | 570 | 733: 304 followed up from wave 1 | 808 out of which 426 have information in wave 3 and some information from either wave 1 and/or wave 2 (50 from wave 1 only; 209 from wave 2 only; 167 from waves 1 and 2) |
| Response Rate | NA* | 38% | 41.88% |

**In view of the urgency and the setting, we could not calculate the response rate because we failed to check how many refused to fill the survey, but refusals were estimated b our staff to be “low”.*
