# Supplementary material for: Unveiling the neglected role of the intensity of acute stress disorder in the prediction of full- and sub-threshold posttraumatic stress disorder: looking beyond the diagnosis
Source: Soc Psychiatry Psychiatr Epidemiol. 2024 Dec 31;60(5):1125–33. doi: 10.1007/s00127-024-02805-z (PMC12119768; doi:10.1007/s00127-024-02805-z)
Supplement: Supplementary file 2 — Supplementary Material 2 [file 127_2024_2805_MOESM2_ESM.docx]

**Title:** Unveiling the Neglected Role of the Intensity of Acute Stress Disorder in the Prediction of full- and sub-threshold posttraumatic stress disorder: Looking Beyond the Diagnosis.

**Journal of Social Psychiatry and Psychiatric Epidemiology**

**Authors names and affiliations:** Elie G. Karam^a,b,c^. Josleen Al Barathie^a^, Hani Dimassi^d^, Franco Mascayano^e,f^, Andre Slim^a^, Aimee Karam^a,b,c^, George Karam^a,b,c^, Katherine M. Keyes^e^, Ezra Susser^e,f^, Richard Bryant^h^.

a Institute for Development, Research, Advocacy and Applied Care (IDRAAC), Beirut, Lebanon

b Department of Psychiatry and Clinical Psychology, University of Balamand Faculty of Medicine, Beirut, Lebanon

c Department of Psychiatry and Clinical Psychology, St George Hospital University Medical Center, Beirut, Lebanon

d School of Pharmacy, Lebanese American University, Beirut, Lebanon

e Department of Epidemiology, Columbia University Mailman School of Public Health, New York, NY, United States

f New York State Psychiatric Institute, New York, NY, United States

h School of Psychology, University of New South Wales, NSW 2052, Sydney, Australia

**Corresponding Author:**

Email: [egkaram@idraac.org](mailto:egkaram@idraac.org)

Supplementary Table 2: NPV and PPV of ASD at 9-15 days after trauma and 21-27 days after trauma and PTSD 6-7 months later: Variations in Total Sample, Full Threshold DSM-5 PTSD Diagnosis and Subthreshold “Majority”, and Full Threshold DSM-5 PTSD Diagnosis and Subthreshold “Six Plus”

|  | N | ASD Diagnosis | | ASD Diagnosis and ASDS Score | |
| --- | --- | --- | --- | --- | --- |
|  |  |  |  | Low intensity | High intensity |
| **Full Threshold DSM-5 PTSD Diagnosis + Subthreshold “Majority”** | | | | | |
| NPV 9-15 days after trauma | 100 | 76 | | 79.57 | 28.57 |
| PPV 9-15 days after trauma | 86 | 55.81 | | 36.84 | 61.19 |
| NPV 21-27 days after trauma | 240 | 67.5 | | 67.8 | 50 |
| PPV 21-27 days after trauma | 105 | 76.19 | | 68.75 | 79.45 |
| **Full Threshold DSM-5 PTSD Diagnosis + Subthreshold “Six Plus”** | | | | | |
| NPV 9-15 days after trauma | 100 | 78 | 80.65 | | 42.86 |
| PPV 9-15 days after trauma | 86 | 61.63 | 36.84 | | 68.66 |
| NPV 21-27 days after trauma | 240 | 67.08 | 67.37 | | 50 |
| PPV 21-27 days after trauma | 105 | 78.1 | 68.75 | | 82.19 |

^*High/low intensity defined as cut-off above or below 58.^
