# Supplementary material for: Unveiling the neglected role of the intensity of acute stress disorder in the prediction of full- and sub-threshold posttraumatic stress disorder: looking beyond the diagnosis
Source: Soc Psychiatry Psychiatr Epidemiol. 2024 Dec 31;60(5):1125–33. doi: 10.1007/s00127-024-02805-z (PMC12119768; doi:10.1007/s00127-024-02805-z)
Supplement: Supplementary file 3 — Supplementary Material 3 [file 127_2024_2805_MOESM3_ESM.docx]

**Title:** Unveiling the Neglected Role of the Intensity of Acute Stress Disorder in the Prediction of full- and sub-threshold posttraumatic stress disorder: Looking Beyond the Diagnosis.

**Journal of Social Psychiatry and Psychiatric Epidemiology**

**Authors names and affiliations:** Elie G. Karam^a,b,c^. Josleen Al Barathie^a^, Hani Dimassi^d^, Franco Mascayano^e,f^, Andre Slim^a^, Aimee Karam^a,b,c^, George Karam^a,b,c^, Katherine M. Keyes^e^, Ezra Susser^e,f^, Richard Bryant^h^.

a Institute for Development, Research, Advocacy and Applied Care (IDRAAC), Beirut, Lebanon

b Department of Psychiatry and Clinical Psychology, University of Balamand Faculty of Medicine, Beirut, Lebanon

c Department of Psychiatry and Clinical Psychology, St George Hospital University Medical Center, Beirut, Lebanon

d School of Pharmacy, Lebanese American University, Beirut, Lebanon

e Department of Epidemiology, Columbia University Mailman School of Public Health, New York, NY, United States

f New York State Psychiatric Institute, New York, NY, United States

h School of Psychology, University of New South Wales, NSW 2052, Sydney, Australia

**Corresponding Author:**

Email: [egkaram@idraac.org](mailto:egkaram@idraac.org)

Supplementary Table 3 Sensitivity and Specificity of ASD at 9-15 days after trauma and 21-27 days after trauma and PTSD 6-7 months later: Variations in Total Sample, Full Threshold DSM-5 PTSD Diagnosis and Subthreshold “Majority”, and Full Threshold DSM-5 PTSD Diagnosis and Subthreshold “Six Plus”

|  | N | ASD Diagnosis | ASD Diagnosis and ASDS Score | |
| --- | --- | --- | --- | --- |
|  |  |  | Low intensity | High intensity |
| **Full Threshold DSM-5 PTSD Diagnosis + Subthreshold “Majority”** | | | | |
| Sensitivity: 9-15 days after trauma | 72 | 66.67 | 26.92 | 89.13 |
| Specificity: 9-15 days after trauma | 114 | 66.67 | 86.05 | 7.14 |
| Sensitivity 21-27 days after trauma | 158 | 50.63 | 24 | 96.55 |
| Specificity: 21-27 days after trauma | 187 | 86.63 | 93.57 | 12.5 |
| **Full Threshold DSM-5 PTSD Diagnosis + Subthreshold “Six Plus”** | | | | |
| Sensitivity: 9-15 days after trauma | 75 | 70.67 | 28 | 92 |
| Specificity: 9-15 days after trauma | 111 | 70.27 | 86.21 | 12.5 |
| Sensitivity 21-27 days after trauma | 161 | 50.93 | 23.76 | 96.67 |
| Specificity: 21-27 days after trauma | 184 | 87.5 | 93.53 | 14.29 |

^High/low intensity defined as cut-off above or below 58.^
