# Supplementary material for: Unveiling the neglected role of the intensity of acute stress disorder in the prediction of full- and sub-threshold posttraumatic stress disorder: looking beyond the diagnosis
Source: Soc Psychiatry Psychiatr Epidemiol. 2024 Dec 31;60(5):1125–33. doi: 10.1007/s00127-024-02805-z (PMC12119768; doi:10.1007/s00127-024-02805-z)

**Title:** Unveiling the Neglected Role of the Intensity of Acute Stress Disorder in the Prediction of full- and sub-threshold posttraumatic stress disorder: Looking Beyond the Diagnosis.

**Journal of Social Psychiatry and Psychiatric Epidemiology**

**Authors names and affiliations:** Elie G. Karam^a,b,c^. Josleen Al Barathie^a^, Hani Dimassi^d^, Franco Mascayano^e,f^, Andre Slim^a^, Aimee Karam^a,b,c^, George Karam^a,b,c^, Katherine M. Keyes^e^, Ezra Susser^e,f^, Richard Bryant^h^.

a Institute for Development, Research, Advocacy and Applied Care (IDRAAC), Beirut, Lebanon

b Department of Psychiatry and Clinical Psychology, University of Balamand Faculty of Medicine, Beirut, Lebanon

c Department of Psychiatry and Clinical Psychology, St George Hospital University Medical Center, Beirut, Lebanon

d School of Pharmacy, Lebanese American University, Beirut, Lebanon

e Department of Epidemiology, Columbia University Mailman School of Public Health, New York, NY, United States

f New York State Psychiatric Institute, New York, NY, United States

h School of Psychology, University of New South Wales, NSW 2052, Sydney, Australia

**Corresponding Author:**

Email: [egkaram@idraac.org](mailto:egkaram@idraac.org)

Supplementary Figure 1: Variation of Negative Predictive Values of ASD 9-15 days after trauma (A) and 21-27 days after trauma (B), and Variation of Positive Predictive Value (PPV) of ASD at 9-15 days after trauma (C) and 21-27 days after trauma (D) predicting the outcome of Subthreshold “Majority” DSM-5 PTSD Diagnosis 6-7 months after trauma

*
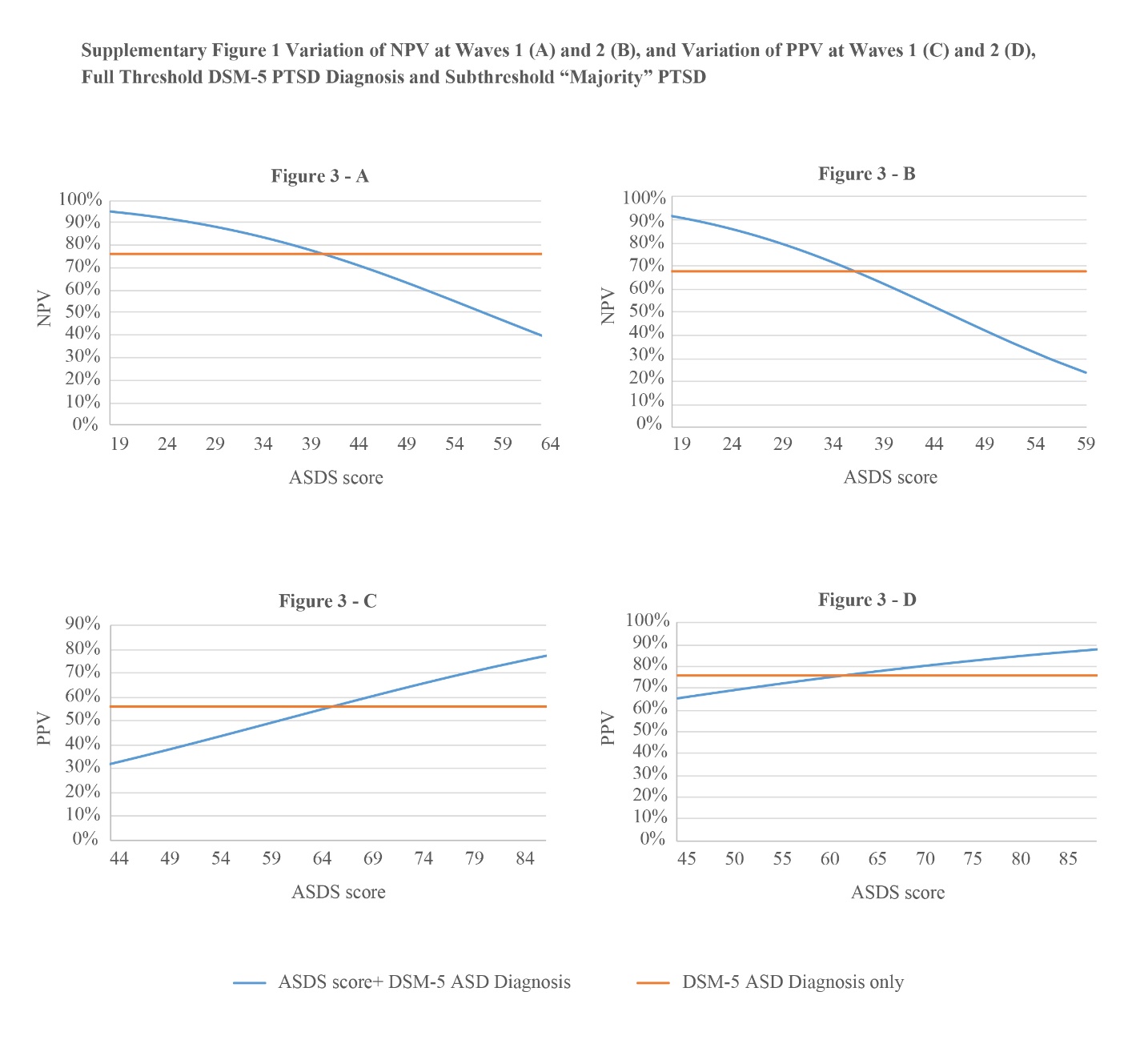
*

Supplementary Figure 2: Variation of Negative Predictive Values of ASD 9-15 days after trauma (A) and 21-27 days after trauma (B), and Variation of Positive Predictive Value (PPV) of ASD at 9-15 days after trauma (C) and 21-27 days after trauma (D) predicting the outcome of Subthreshold “Six Plus” DSM-5 PTSD Diagnosis 6-7 months after trauma


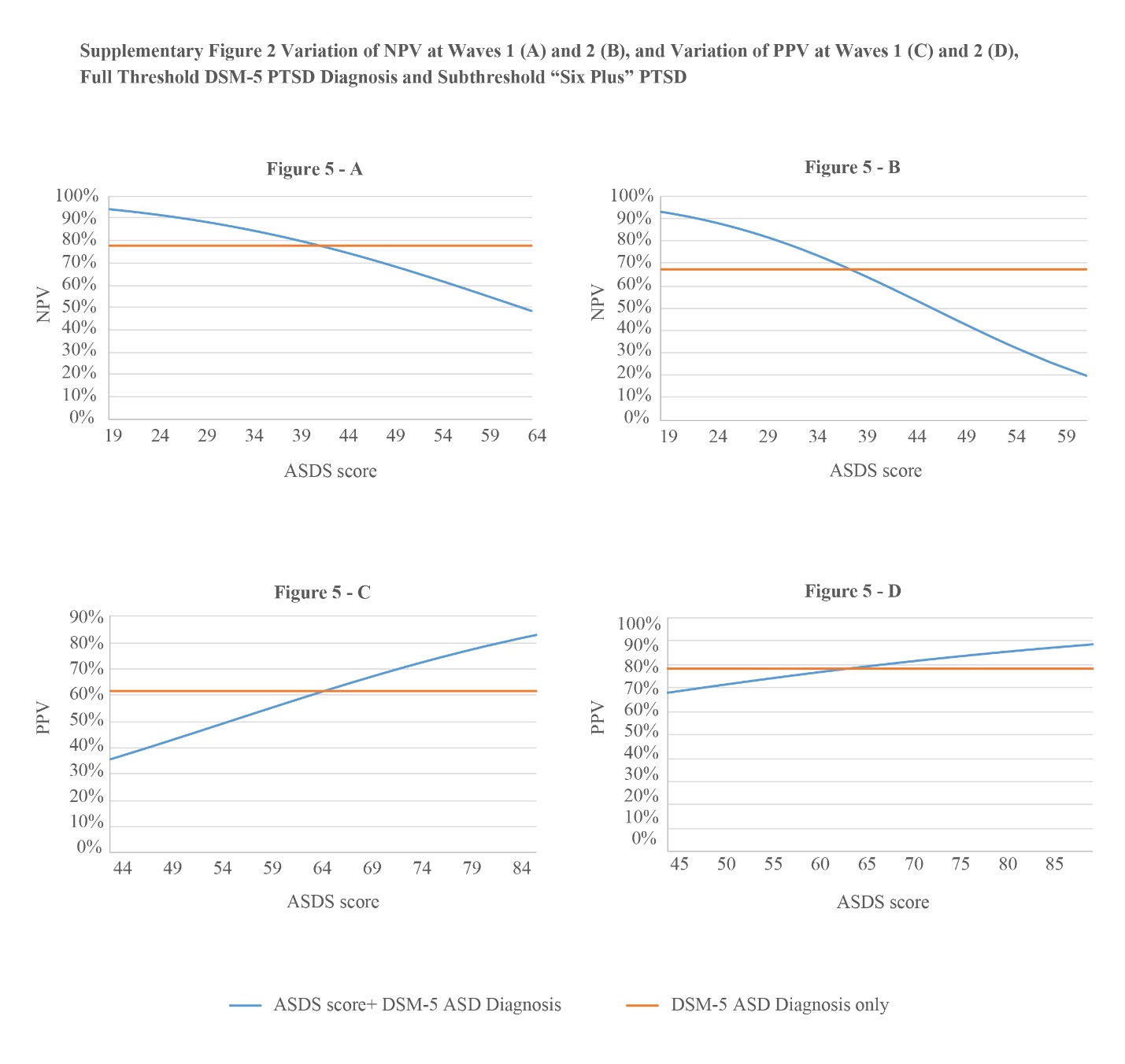

Supplement: Supplementary file 4 — Supplementary Material 4 [file 127_2024_2805_MOESM4_ESM.docx]
